# An experimental online study on the impact of negative social media comments on anxiety and mood

**DOI:** 10.1038/s41598-025-10810-8

**Published:** 2025-07-22

**Authors:** Yuetong Ai, Adrian von Mühlenen

**Affiliations:** Department of Psychology, University of Warwick, Coventry, CV4 7AL UK

**Keywords:** Social media, Anxiety, Negative comment, Mood state, Cyberpsychology, Psychology, Quality of life

## Abstract

Social media has become the mainstream communication platform, offering unprecedented convenience, but its anonymity can also encourage negative interactions. This study investigates how negative comments on social media affect adults’ anxiety and mood. In an experimental study involving 128 adult participants (85 female, 43 male), individuals were asked to share blog posts on a simulated internet forum. Subsequently, they were exposed to either negative, neutral, or positive comments, and their mood and anxiety levels were measured using validated scales. Results showed that negative comments significantly increased anxiety and decreased mood compared to neutral or positive comments, while gender did not show any significant effects. Younger adults reported stronger anxiety responses to negative comments than older adults, suggesting heightened sensitivity among younger users. These findings highlight the psychological impact of negative social media comments on adults and underscore the importance of strategies for managing online negativity. This research expands our understanding of social media’s effects on adult mental health.

## Introduction

With the development of modern science and technology, social media has dramatically changed how people interact worldwide, becoming a mainstream channel of human communication. Facebook, Twitter, Instagram, and TikTok have grown rapidly, reaching over five billion users worldwide^1^ and reshaping public discourse, communication, and information sharing. The popularity of social media has brought about an accessible means of information sharing, challenging the dominance of more traditional media such as newspapers, radio or TV. Individuals and organizations can now influence public opinion, creating social movements in ways that were not possible before. For example, the Arab Spring is a well-known event that mobilized social forces promoting social change through media like Twitter or Facebook^2^. This mode of communication enabled social media to transcend its technical product positioning. Social media has increasingly influenced cultural, economic, and political discourse in modern society^3^. Social media allows relatively free speech and provides rich learning resources to promote equality in education; the Open Educational Practice website is a good example^4,5^. Additionally, social media provides a platform for marginalized groups to make their voices heard, enabling them to bring societal changes^6^. It also creates a brand community for businesses to foster customer trust and loyalty^7^.

While social media facilitates global connectivity and information exchange, it also presents significant psychological risks, such as increased exposure to negative interactions and cyberbullying^8,9^. The overuse of social media can also lead to user addiction, making them feel lonely^10^ and affecting their emotional health^11^. Unwanted abstinence from or prevented access to social media can also lead to feelings of boredom and cravings^12^. Braghieri et al.^13^ conducted a quasi-experimental study by gradually introducing Facebook across colleges, allowing them to assess its impact on student mental health. Their findings revealed that the platform’s rollout led to a significant decline in mental wellbeing, particularly among women, and was linked to increased use of mental health services and decreased academic performance. They attributed these effects to the role of Facebook in promoting unfavorable social comparisons—highlighting the psychological costs of harmful or comparative content on social media.

Hunt et al.^14^ conducted a randomized controlled trial in which college students were assigned either to a group that reduced their social media use or to a group that maintained their usual habits. Participants who limited their use reported significantly lower levels of loneliness and depression compared to the control group, providing further evidence that reducing exposure to potentially harmful social media content can benefit emotional wellbeing. While these studies establish a general link between social media use and mental health, relatively little is known about the specific emotional impact of negative user comments. For instance, Chen et al.^15^ found that participants exhibited stronger negative emotional responses to hostile, uncivil disagreements than to polite ones, suggesting that the tone and delivery of comments on social media may directly influence users’ emotional experiences.

This paper will focus on the negative effect that social media provides on cyberbullying. More specifically, on social media, users’ real identities are not easily directly linked to the content they post. This promotes their perception of anonymity and generally makes them feel less morally sensitive^16,17^. When moral constraints are weakened, users feel less inhibited. The online disinhibition phenomenon increases the likelihood of producing negative comments on social media^18^. These negative comments, including – but not limited to – sarcasm, personal attacks, malicious provocations and discrimination, can often contribute to psychological distress. Studies indicate that cyberbullying on social media correlates with increased psychological stress and depressive symptoms^19,20^. In addition, negative comments can aggravate an individual’s symptoms. Adolescents with anxiety disorders often have low self-esteem, which makes them more sensitive to comments on social media^21^. Even minor negative comments can impact their self-esteem, which is going to lead to more anxiety, possibly creating a vicious cycle. Fortunately, with the publication of these studies, more attention has been paid to the impact of negative comments on adolescents, and interventions have been proposed.

Most previous research has examined the impact of negative comments on adolescents, yet there is limited understanding of these effects in adults^22,23^. This study uses an experimental method to address this gap by evaluating the effects of negative social media comments on adult anxiety and mood, hypothesizing that such comments will elevate anxiety and reduce mood. Previous research has also shown that women are more likely to feel anxious than men^24,25^. Therefore, it is predicted that female participants will be more affected by negative comments than male participants.

## Method

### Participants

A power analysis using G-Power^26^ indicated that a minimum of 128 participants would be required to detect a medium effect size (f = 0.25) in a 2 (Gender) x 3 (Comment Type) between-subjects ANOVA, with α = 0.05 and power (1 – β) = 0.80. To account for potential attrition, 142 adult participants were recruited via Prolific’s online research platform (www.prolific.com). Only English-speaking participants were chosen to ensure they understood the written blogs and comments well. All participants gave written informed consent and were compensated £7 for participation. Twelve participants chose to withdraw their data at the end of the study, and one participant failed the attention checks, leaving 129 participants (85 females, 43 males, and 1 “other”), all over 18 years old (M = 37.0, SD = 12.73). The study was approved by the Ethics Committee of the Department of Psychology at the University of Warwick, and all methods were performed in accordance with the University’s guidelines and regulations. The study design, materials, and hypotheses were pre-registered on the Open Science Framework^27^.

### Design, stimuli, and measures

The study used a between-subjects design with two independent variables: comment type (negative, neutral, or positive) and participant gender. While participants were randomly assigned to a comment type condition, gender served as a quasi-experimental factor. This design aims to investigate how the two variables affected participants’ mood states and anxious levels, examining their individual effects and interactions.

**Fig. 1:**
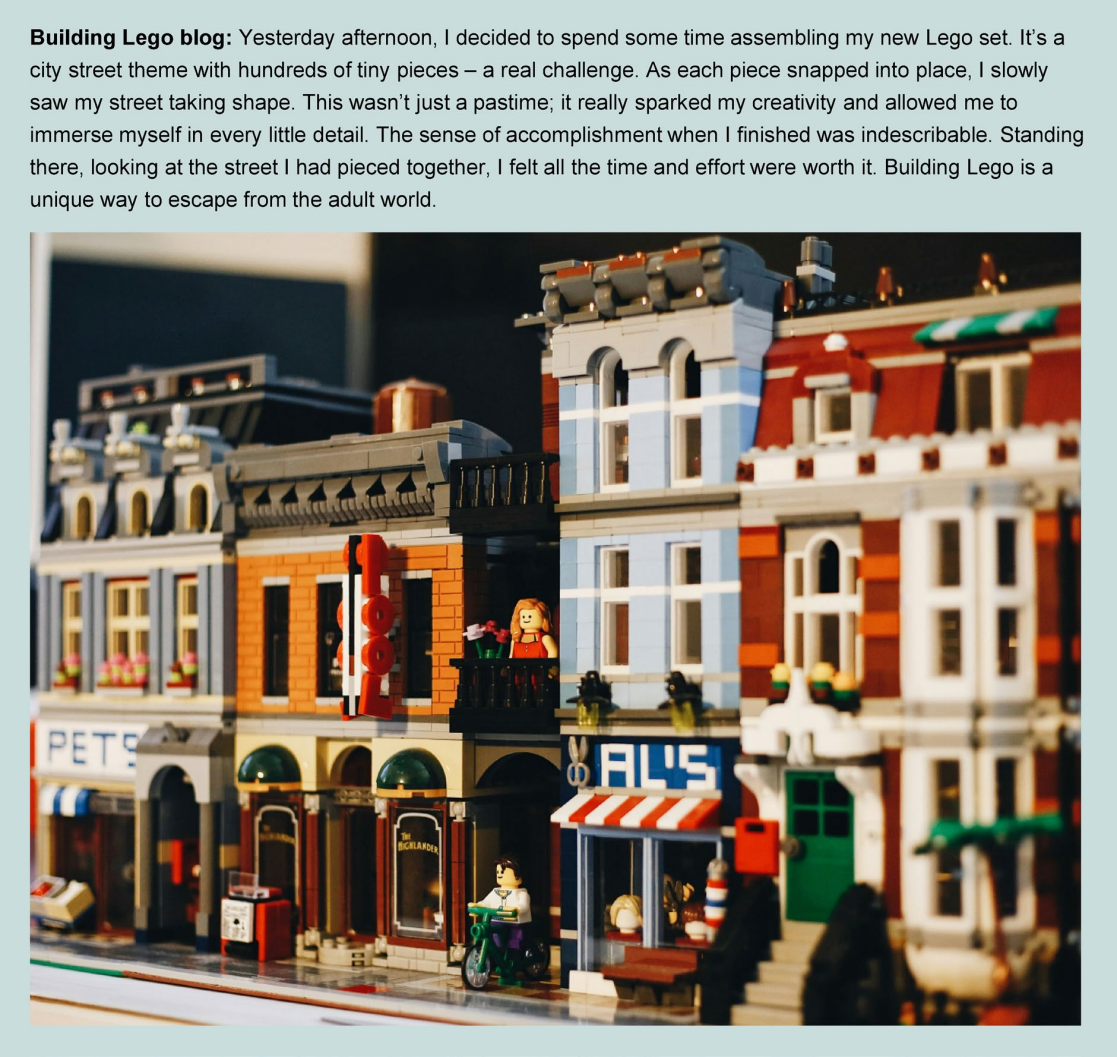
An example showing the Building Lego blog with its corresponding image. From Alphacolor (2017). [White and multicolored building scale mode]. Unsplash. https://unsplash.com/photos/ipmwlGIXzcw.

We used AI-generated blogs to maintain better experimental control over content length and quality rather than having participants write their own blogs. This approach also minimized variability due to differences in individual writing ability and effort. The blogs were generated using ChatGPT, with a first prompt providing a brief description of the study’s purpose and design. The second prompt asked to generate a blog on the given topic. The third, fourth and final prompt asked to generate ten positive, ten neutral, and ten negative comments, respectively. The full prompts are available on the Open Science Framework^27^ (file “ChatGTP_Prompts.pdf”). In total, eight blogs (short texts of 75–100 words) were generated, each underlined by a picture that matched the blog’s theme (see Fig. 1 for an example).

The blogs were presented in pairs, and participants had to choose one topic each time for their blog. The option to choose was primarily introduced to enhance participants’ motivation and engagement with the material (e.g.,^28^). The topics were chosen by the authors to be of interest to the broader public and included the following pairs: shopping/building Lego, gaming/drinking, gardening/baking, and upscale dinner/trip. The eight pictures were taken from a freely usable image source on the internet (www.unsplash.com). They showed people walking in an antique shopping arcade^29^, buildings built in Lego^30^, two people playing on videogame consoles^31^, a close-up image of a cocktail drink^32^, a person watering garden plants^33^, a raspberry cake^34^, a salmon and vegetable dish^35^, or people walking in an alleyway^36^. Each topic choice was followed by the corresponding ten comments, which were either consistently negative, neutral, or positive. For example, a negative comment would be, “These Lego models look really childish; don’t you think you’re too old for this?”, a neutral comment would be, “Hi, where do you usually buy your Lego sets?”, and a positive comment would be, “You’re so talented with Lego, it’s truly impressive!”. Thus, each participant viewed 40 comments out of a total of 240 (i.e., 8 × 3 × 10) comments. Note that there was an error affecting some neutral comments, where about a third of them were preceded by a made-up username (e.g., “@roomie”). However, we don’t believe this had much impact on the overall results. Before the study, we conducted a simple manipulation check by showing the blogs and comments to two naive volunteers. They were asked to confirm (yes or no) whether the comments conveyed the appropriate sentiment for their category. None of the comments were changed or replaced as a result of this check. The content of the blog posts, the corresponding images, and the comments are fully available on the Open Science Framework^27^ (see “Procedure.docx” and JPG files).

The participants’ mood states were measured using Mayer and Gaschke’s^37^ Brief Mood Introspection Scale (BMIS). The scale includes 16 adjectives describing different moods, such as ”lively”, ”happy”, or ”gloomy”. Participants were asked to rate each word on a scale from 1 (“definitely do not feel”) to 4 (“definitely feel”) to indicate how well the words described their current mood. Anxiety level was measured with Spielberger et al.’s^38^ State-Trait Anxiety Inventory (STAI original Form X-1), which is suitable for measuring anxiety across different cultures^39^. Only the state scale (STAI-S) was used, which contains 20 short statements, such as “I feel calm”, “I feel secure”, or “I am tense”. Participants had to rate each statement on a four-point scale from 1 (“not at all”) to 4 (“very much”) to indicate how they felt right at this moment.

### Procedure

The experiment started by explaining the purpose of the study, the data handling, and the participant rights, followed by the consent form and questions about age and gender. In the first part of the experiment, participants were asked to imagine that they were a blogger with a hundred thousand followers who would update their blog regularly every day. They further had to imagine that they were sitting in front of their computer and that they would, with a happy heart, share something exciting. Participants then pressed “continue” before seeing the first two topics with the corresponding blogs and images. They were told they did not need to write anything; they just had to select the topic that interested them more, and the chosen blog would be automatically shared. Please note that participants were unaware that their choice had no effect whatsoever; its purpose was only to make them feel more immersed. Participants were then asked to wait while other users (apparently) were viewing and commenting on their blog. After a few seconds, a new page appeared showing the comments on their blog, which were either all negative, neutral, or positive, depending on their comment type condition. They were asked to read the comments carefully, as they may get questions related to them later. This procedure of choosing and reading comments was repeated four times.

In the second part of the experiment, participants completed the BMIS and the STAI-S. Both questionnaires contained two attention checks, where participants were asked to select a particular answer (e.g., “select 3”). After completing the questionnaires, to neutralize any negative emotions potentially caused by the comments, participants were shown a funny 90-second video on YouTube^40^. At the end, participants were debriefed and redirected to Prolific to be compensated for their participation.

### Data analysis

BMIS mood scores were calculated separately for each participant by adding the responses of all pleasant items (e.g., “happy”) and the reversed responses of all unpleasant items (e.g., “gloomy”; for item coding, see^37^). The sums were then divided by sixteen, the number of items, to have the final scores again ranging from 1 to 4 (i.e., from “not at all” to “very much”). Individual STAI-S anxiety scores were calculated by adding all negative items (e.g., “I am tense”) and reversed positive items (e.g., “I feel calm”, for item coding, see^41^). The sums were again divided by the number of items to have the final scores ranging from 1 to 4. The data was analyzed using JASP^42^.

## Results

### Blog choices

Although the primary purpose of allowing participants to choose blog topics was to enhance their engagement with the material, we first examined their choices and whether these affected their anxiety or mood. The number of participants selecting each option was as follows: shopping (*n* = 49) versus building Lego (*n* = 80), gaming (*n* = 63) versus drinking (*n* = 66), gardening (*n* = 60) versus baking (*n* = 69), and upscale dinner (*n* = 23) versus trip (*n* = 106). Independent-samples t-tests conducted for each pair revealed no significant effects on anxiety (all *p* >.078), pleasant mood, or the other three mood sub-scores (all *p* >.062), indicating that anxiety and mood were not influenced by participants’ specific topic choices.

### State-trait anxiety inventory (STAI)

**Fig. 2:**
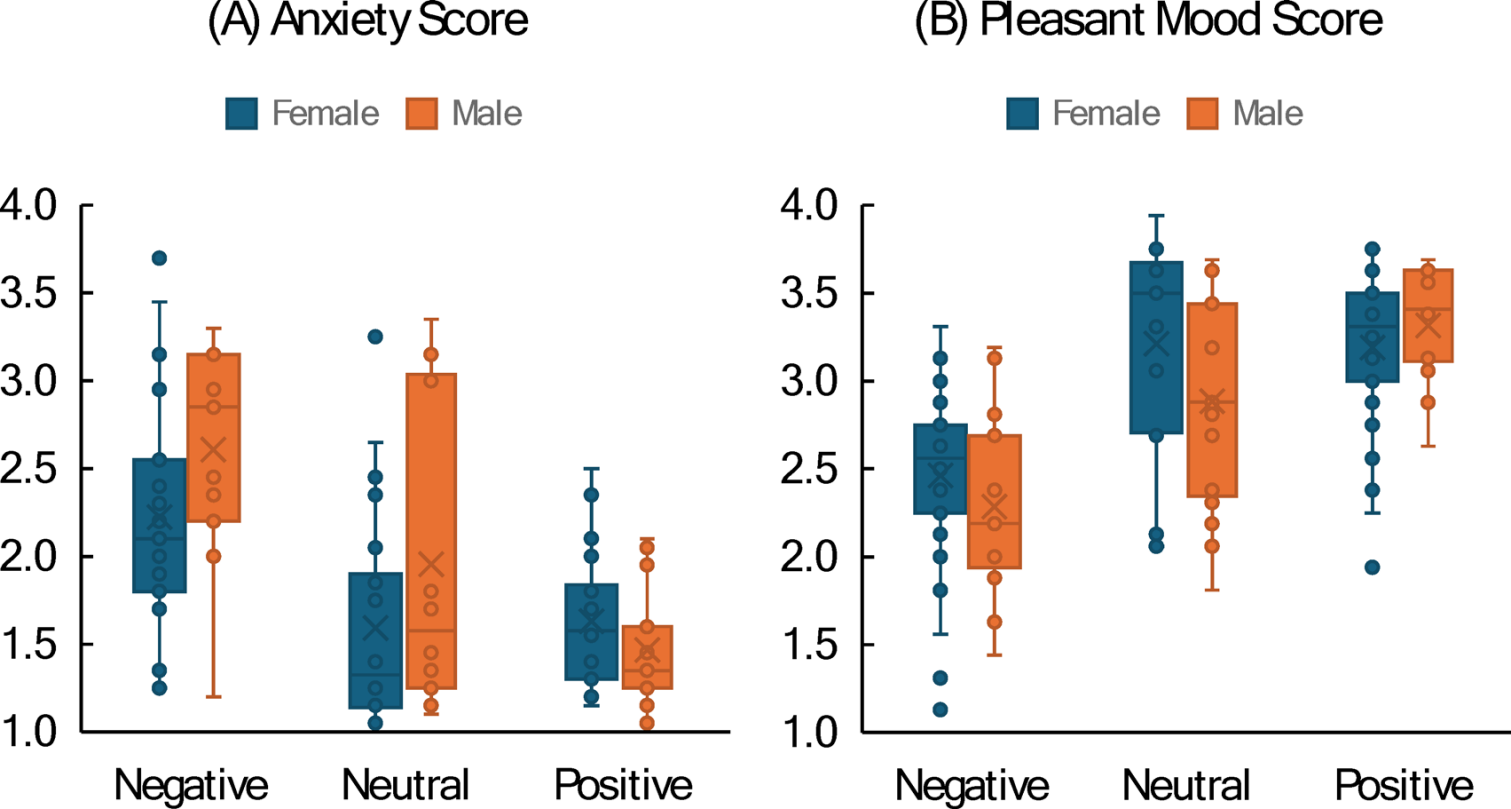
Boxplots showing (**A**) anxiety score and (**B**) pleasant mood score as a function of comment type (negative, neutral, positive) with separate distributions for female (blue) and male (orange) participants. Bars represent the interquartile range; whiskers indicate the full data range.

Participants selecting “prefer not to say” in one or more responses (*n* = 7) were excluded from the following analysis. The data from one participant who selected “other” for gender was excluded because the group size was too small for meaningful analysis. Figure 2A shows the anxiety scores averaged across participants for each combination of Gender and Comment Type.

A 2 × 3-way univariate ANOVA with the between-subject factors Gender (male, female) and Comment Type (negative, neutral, positive) revealed a significant main effect of Comment Type, F(2, 115) = 19.74, *p* <.001, η_p_^2^ = 0.256. Two-tailed post-hoc tests^43^ revealed that negative comments lead to significantly higher anxiety scores than neutral or positive comments (2.42 vs. 1.77 and 1.55, respectively; both *p* <.001, the difference between the latter two was not significant). Because Levene’s test revealed an unequal variance between groups, F(5, 115) = 5.45, *p* <.001, the significant Comment Type effect was confirmed with a Kruskal-Wallis test, H(2) = 26.83, *p* <.001. Figure 2A suggests that male participants receiving negative or neutral comments were more anxious than female participants. However, the Gender main effect, F(1, 115) = 2.77, *p* =.099, η_p_^2^ = 0.024, and the interaction between Gender and Comment Type, F(2, 115) = 2.37, *p* =.098, η_p_^2^ = 0.040, were both only marginally significant.

During a preliminary inspection of the data, it was discovered that the Prolific sample had an unexpectedly widespread age range (18 to 73 years). Given the evidence that older and younger adults differ in their social media use, such as frequency, purpose, and preferred platforms^44,45^, we decided to conduct further exploratory analyses to examine age-related effects (note that these were not pre-registered). Participants were split by the median (Mdn = 35 years) into two groups: a younger age group (*n* = 61, M = 26.72, SD = 4.23) and an older age group (*n* = 60, M = 48.13, SD = 9.37). Age Group (younger, older) was then added as an additional factor to the current design to test whether anxiety levels differed by age. The 2 × 2 × 3-way ANOVA with the factors Age Group, Gender, and Comment Type revealed a significant main effect of Age Group, F(1, 109) = 6.73, *p* =.011, η_p_^2^ = 0.058, due to younger participants reporting to be more anxious than older participants (2.07 vs. 1.77, respectively). None of the other effects involving Age Group reached significance (all *p* >.200).

### Brief mood introspection scale (BMIS)

Participants selecting “prefer not to say” in one or more responses (*n* = 5) were excluded from the analysis. The data from the participant selecting “other” for gender (*n* = 1) was not included. The mood scores averaged across the remaining 123 participants are shown in Fig. 2B.

A 2 × 3-way ANOVA with the between-subject factors Gender and Comment Type revealed a significant main effect of Comment Type, F(2, 117) = 28.13, *p* <.001, η_p_^2^ = 0.325: Two-tailed post-hoc tests revealed that negative comments lead to significantly lower mood scores than neutral or positive comments (2.37 vs. 3.05 and 3.25, respectively), mirroring the reversed pattern of the anxiety scores (compare Fig. 2A and B). Note that Levene’s test was nonsignificant for mood (*p* =.103), indicating sufficient equality of variance between groups. Gender effects were not significant (all *p* >.182). Adding Age Group as an additional factor to the ANOVA revealed a significant Age main effect, F(2, 111) = 5.10, *p* =.026, η_p_^2^ = 0.044, due to younger participants reporting less pleasant mood than older participants (2.79 vs. 3.01, respectively). None of the interactions involving Age Group reached significance (all *p* >.090).

**Fig. 3:**
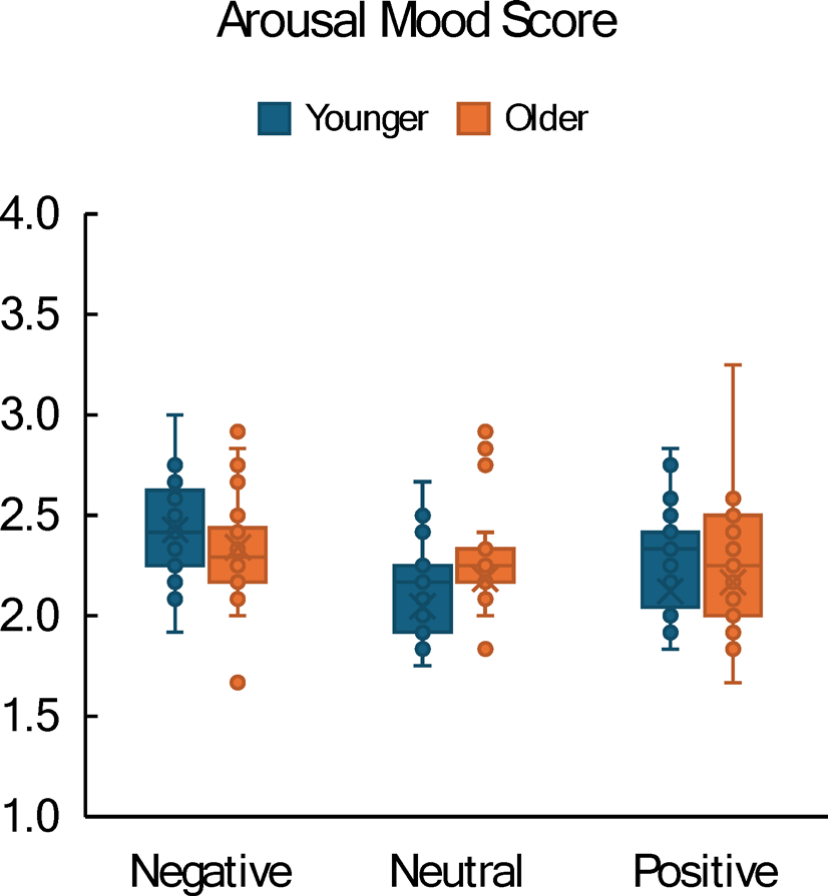
Boxplots showing arousal mood score as a function of comment type (negative, neutral, positive) with separate distributions for younger (blue) and older (orange) participants. Bars represent the interquartile range; whiskers indicate the full data range.

Mayer and Gaschke^37^ proposed that three other sub-scores can be computed from the BMIS (in addition to pleasant versus unpleasant mood). For example, twelve of the 16 BMIS items can also be used to represent arousal (10 items, e.g., “lively”, “sad”) versus calm mood (2 items: “tired”, “calm”). Although these sub-scores were not included in the pre-registered analysis plan, they were explored in a post-hoc analysis. Individual arousal scores were calculated by summing up arousal items and reversing calm items, and by dividing the sum by the number of items. The 2 × 3 ANOVA with Gender and Comment Type revealed significant main effects for Gender, F(1, 117) = 6.15, *p* =.015, η_p_^2^ = 0.050, and Comment Type, F(1, 117) = 4.69, *p* =.012, η_p_^2^ = 0.073: Male participants reported overall more arousal than female participants (2.39 vs. 2.26, respectively), and negative comments lead to more arousal than neutral comments (2.44 vs. 2.24). Adding Age Group to the ANOVA revealed a significant interaction between Age Group and Comment Type, F(1, 111) = 4.11, *p* =.019, η_p_^2^ = 0.069: As can be seen in Fig. 3, older participants were overall less aroused by negative or positive comments (compared to neutral comments) than younger participants (0.03 or −0.05 vs. 0.29 or 0.18, respectively).

The BMIS can also represent positive versus tired mood (7 items) and negative versus relaxed mood (6 items). For positive versus tired mood, the pattern mirrored that of pleasant mood, with significant main effects for Comment Type, F(2,112) = 16.35, *p* <.001, η_p_^2^ = 0.226, and Age Group, F(1,112) = 6.01, *p* =.016, η_p_^2^ = 0.051. In contrast, the negative versus relaxed mood pattern resembled the inverse of pleasant mood, with significant main effects for Comment Type, F(2,114) = 27.24, *p* <.001, η_p_^2^ = 0.323, and Age Group, F(1,114) = 4.23, *p* =.042, η_p_^2^ = 0.036. However, for these sub-scores, the interaction between Age Group and Comment Type did not reach significance (both *p* >.057).

## Discussion

This study was designed to explore how negative comments on social media affect the mood state and anxiety levels of adults of different genders. The results showed that negative comments significantly increased anxiety and caused more unpleasant moods compared with positive or neutral comments, with gender showing only a marginal effect on anxiety and no significant influence on unpleasant moods. What we didn’t expect was a wide range of ages, so we included age as a factor and found that younger people were more likely to have anxiety and negative moods than older people. These results align with previous findings on adolescents^19^, but extend the evidence to adults, underscoring the potential psychological impacts of negative social media interactions across age groups. Arousal levels were also affected by age, gender, and comment type. Male participants reported greater overall arousal than females, and younger participants exhibited stronger arousal responses to both negative and positive comments. These findings mirror those of anxiety, which fits with the well-established concept that anxiety is associated with increased arousal, both subjectively and physiologically^46^.

Subdividing the adult sample revealed that the adverse psychological effects of social media persisted among younger adults but appeared to diminish in older adults. Younger adults may still form their self-identity and social roles^47,48^, emphasising their online image and perceived social attractiveness. This heightened concern can make them more vulnerable to negative emotions in response to criticism or competitive interactions^49,50^. Another possible explanation is that younger adults may be more affected by the psychological construct of Fear of Missing Out (FOMO), which can intensify stress responses to social comparisons and critical feedback^51^ and increase feelings of jealousy^52^. For example, when they see comments like “The products I purchase are more luxurious than yours,” they experience increased jealousy, which can lead to anxiety^53^.

The subdivision of age in our study aligns with prior research suggesting that adults tend to be more psychologically resilient than adolescents^54,55^. This may reflect a gradual, linear increase in mental toughness across the lifespan, supporting the need for future studies to examine age-related effects in greater detail^56^. These findings suggest that intervention strategies should be tailored to specific age groups. For example, younger adults may benefit from cognitive reappraisal training to better manage social comparisons and reinterpret negative evaluations on social media^57^. For older adults (35 + years), enhancing digital literacy could reduce the risk of misinterpreting online interactions, particularly through an improved understanding of algorithmic filtering and content presentation^58^.

Although prior research has found gender differences in anxiety responses, with women generally reporting higher anxiety levels^24,25^, our study observed slightly higher anxiety ratings among men in response to negative social media comments. This result may reflect gender-specific pressures within the context of online environments. One possible explanation is that men may experience greater pressure to conform to social expectations around emotional restraint and interpersonal conduct, particularly when attempting to maintain traditional markers of masculinity, such as projecting a positive body image^59–61^. As a result, negative evaluations may pose a stronger threat to men’s self-esteem, leading to elevated anxiety. Furthermore, men may interpret such evaluations as a challenge to their social standing, increasing the perceived risk of exclusion and triggering heightened social stress^62^.

In contrast to the STAI, which explicitly measures anxiety, the BMIS provides a composite assessment of the overall mood state by aggregating multiple mood components. However, this aggregation can produce a “cancellation effect” that may obscure underlying gender differences. When facing negative evaluations, individuals may experience a mix of emotional responses—such as anxiety, anger, and disappointment—whose intensity and dominance may differ by gender. For instance, women may be more likely than men to experience sadness in such situations^63^. If one participant reports high anxiety and low sadness, and another the reverse, their overall mood scores may converge, masking actual emotional differences.

Moreover, our experiment did not record individual differences among participants. For example, having prior social support and high self-efficacy can lead to better mood and less anxiety^64,65^. Personality traits and emotional intelligence, to a certain extent, determine individuals’ coping abilities and strategies^66,67^. If men and women had similar levels in these areas, the gender difference might be cancelled out, which in line with overall explains why gender has only a very subtle effect.

Although we employed a highly realistic experimental design, enhancing the external validity of the results—meaning that most people would react similarly to negative comments as the sample in our experiment^68^. There are still many designs that need improvement. Firstly, participants may have realized they were part of an experiment, which could have led to the observer effect, where participants were aware that the experimenter would observe their data and change their responses^69^. Secondly, the comments received by participants were all generated by ChatGPT. Although these comments were designed to be negative, neutral, or positive, they may not fully reflect the complexity and diversity of comments in real social media environments. Real online comments also include non-text elements like emojis^70^. Thirdly, our participants were mainly adult native English speakers, likely from Western cultural backgrounds. Different cultures use social media differently^71^. Individuals from specific cultural backgrounds may be more inclined to gain confidence through social media, making them more sensitive to the influence of negative comments.

This study’s reliance on simulated social media comments, while valuable for experimental control, may not fully capture the complex dynamics of real-world interactions. Future studies could utilize actual social media platforms to increase ecological validity. For example, researchers could ask participants to post something during the experiment on their real Instagram account. More objective data on mood state and anxiety levels could be obtained with physiological indicators or behavioral measurements, such as heart rate, skin conductance responses, and facial expression analysis, combined with self-report measures. To more accurately reflect the social media environment, researchers can collect user comments from real media like Twitter or construct a diverse comment library that includes elements like metaphors and emojis to simulate the complexity of real comments. Moreover, the comments in this study all focused on lifestyle-related themes, leaving it unclear whether the findings can be generalised to other, more social or political themes (e.g., group identity), which may elicit stronger emotional reactions. Finally, recruiting a culturally diverse sample would provide a more comprehensive understanding of how different populations are affected by social media negativity. The current findings underscore the need for social media platforms and mental health organizations to provide resources for managing negative online interactions, potentially through digital literacy programs or features that allow users to moderate their exposure to certain content.

## Data Availability

The datasets generated and analyzed during the current study, along with the corresponding R scripts, are available in the OSF repository at https://doi.org/10.17605/OSF.IO/EF4ND.
